# When Two Roads Intersect: Coexisting Achalasia Cardia and Oesophageal Varices as a Therapeutic Dilemma

**DOI:** 10.7759/cureus.90404

**Published:** 2025-08-18

**Authors:** FNU Nauman, Usman Saleem, Gayathri Jayakumar, Sadia Ikram

**Affiliations:** 1 Gastroenterology and Hepatology, Frimley Health National Health Service Foundation Trust, Frimley, GBR; 2 Internal Medicine, Royal International Medical Center, Abu Dhabi, ARE

**Keywords:** achalasia cardia, botox injections, endoscopic ultrasound (eus), gastroenterology and endoscopy, oesophageal varices

## Abstract

Achalasia is a rare cause of oesophageal dysmotility. This disorder causes impaired relaxation of the lower oesophageal sphincter (LOS), impacting peristalsis and the normal movement of food through the digestive tract, resulting in worsening swallowing difficulty, known as dysphagia. Although various effective treatments are available, like pneumatic dilation, laparoscopic Heller myotomy, and peroral endoscopic myotomy (POEM), patients with portal hypertension may develop oesophageal varices, which complicates treatment due to the heightened risk of severe bleeding.

We present the case of a 45-year-old man with a history of cirrhosis and chronic hepatitis C who developed progressively worsening dysphagia over four months, which led to significant weight loss over this period and prompted further investigation. An oesophagogastroduodenoscopy (OGD) showed a dilated oesophagus with retained food contents and grade II oesophageal varices without any endoscopic red signs. Additionally, a barium swallow and high-resolution manometry were done, which led to the confirmation of the diagnosis of achalasia cardia. Given the high risk of bleeding associated with conventional therapies in the presence of varices, a safer, minimally invasive approach was chosen. Botulinum toxin was carefully injected into the LOS under endoscopic ultrasound (EUS) guidance to avoid the variceal sites and negate the risk of bleeding. This approach allowed targeted treatment of achalasia while minimising the risk of variceal bleeding. The patient experienced significant improvement in dysphagia without complications.

This case highlights the rare and challenging coexistence of achalasia and oesophageal varices. It highlights the need for considering individualised treatment strategies in high-risk individuals.

## Introduction

Achalasia is a primary oesophageal motility disorder. It is a very rare condition, with an annual incidence estimated between 1.07 and 2.2 cases per 100,000 individuals, with prevalence rates estimated between 10 and 15.7 per 100,000 individuals [1]. It is specific to the oesophagus and does not affect any other sites in the gut. This motility disorder of unknown aetiology causes degeneration of the myenteric plexus of the oesophageal wall and affects the normal function of the oesophageal smooth muscle, failing relaxation of the lower oesophageal sphincter (LOS), and absence of peristalsis [2]. If left untreated, achalasia can lead to many complications, including dehydration, weight loss, and malnourishment, which could be detrimental in patients who have other comorbid conditions, such as cirrhosis [1,3,4]. Various treatment modalities are available, including medical therapy such as nitrates and calcium channel blockers, endoscopic treatment such as pneumatic dilatation and peroral endoscopic myotomy (POEM), or surgery [1,5].

Botulinum toxin is a type of neurotoxin that is recommended as a treatment option for achalasia, especially for those with multiple comorbidities. It acts by blocking the release of acetylcholine, a neurotransmitter that causes muscles to contract [5-8]. Although the botulinum toxin injection (BTI) shows lesser therapeutic efficacy compared to pneumatic dilation and myotomy, its superior safety profile, simplified technique, faster patient recovery, and effectiveness in cases of vigorous achalasia, such as achalasia type III as per Chicago classification, have established its value [9-12]. In addition to this, BTI primarily serves as a treatment alternative for patients with substantial health issues (for example, liver cirrhosis, frailty), those awaiting surgery, or individuals unwilling to undergo standard procedures [5-9].

The presence of oesophageal varices prompts difficult decisions in the management of achalasia due to the potential risk of bleeding from varices. This case report highlights that patients at significant risk of bleeding or with oesophageal varices can be considered for endoscopic ultrasound (EUS)-guided BTI [5-7]. 

## Case presentation

A 45-year-old man with a background of chronic hepatitis C complicated by liver cirrhosis and portal hypertension presented with a history of dysphagia and weight loss. He had completed a three-month course of sofosbuvir/velpatasvir for hepatitis C and achieved a sustained virological response (SVR). On presentation, he reported a four-month history of dysphagia and symptoms of reflux, including occasional retrosternal chest pain. Additionally, he had lost approximately four kilograms of weight, secondary to dysphagia and reduced oral intake. 

**Figure 1:**
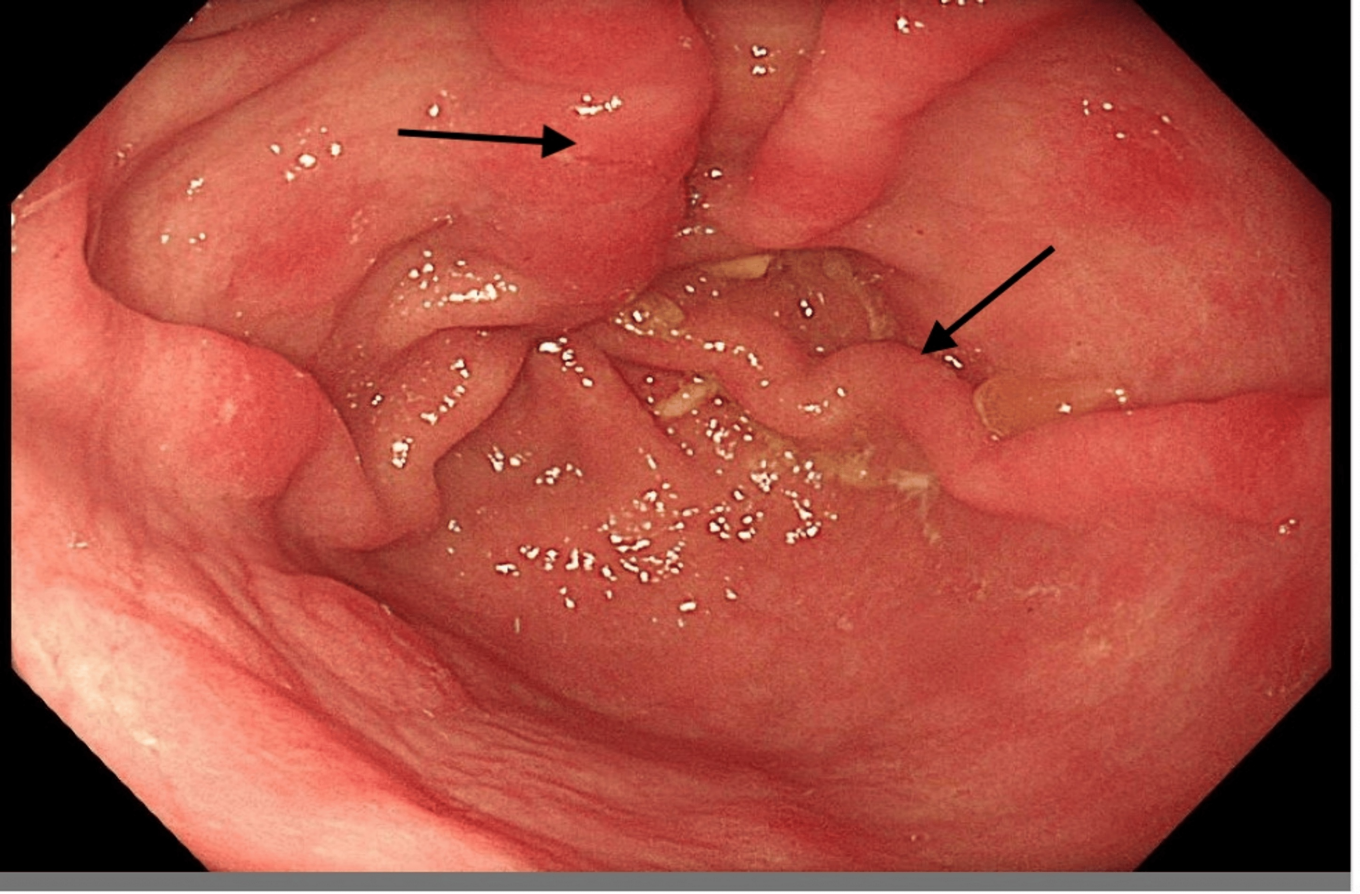
Grade II oesophageal varices (arrows) with dilated oesophagus and pinpoint gastro-oesophageal junction (GOJ)

He underwent an oesophagogastroduodenoscopy (OGD), which revealed a dilated, fluid-filled oesophagus with some food residue along with a well-covered grade-2 oesophageal varices (Figure 1) without any high-risk features.

**Figure 2:**
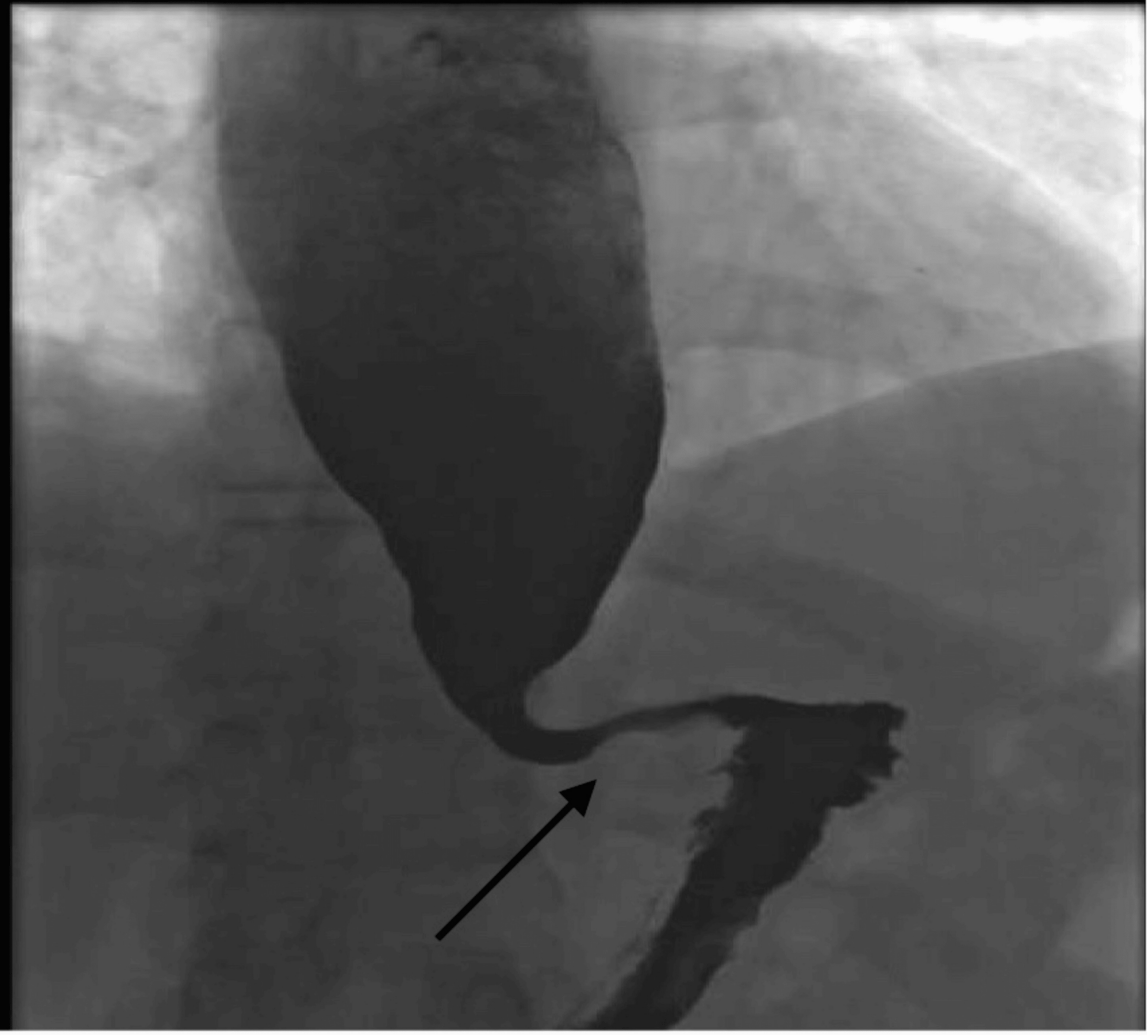
Barium swallow of the same patient revealing a bird beak appearance of the GOJ GOJ: gastro-oesophageal junction

His presenting symptoms prompted further investigation, and he underwent a barium swallow study (Figure 2), which revealed a bird-beak appearance suggestive of achalasia.

**Figure 3:**
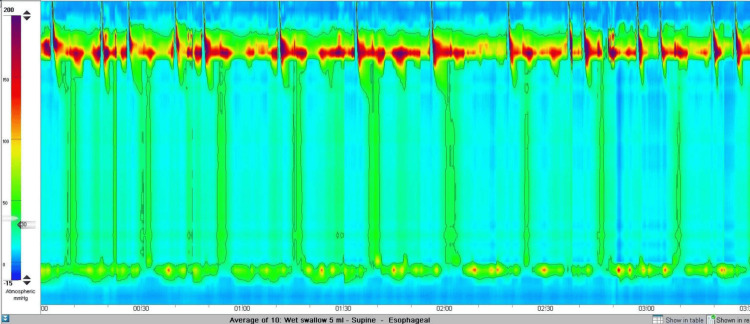
High-resolution manometry showing raised IRP (28.9 mmHg), 90% panoesophageal pressurisation, and no normal peristalsis IRP: integrated relaxation pressure

This prompted a high-resolution oesophageal manometry study. The study showed an integrated relaxation pressure of 28.9 mmHg (Figure 3), along with pan-pressurisation, suggestive of type II achalasia.

**Figure 4:**
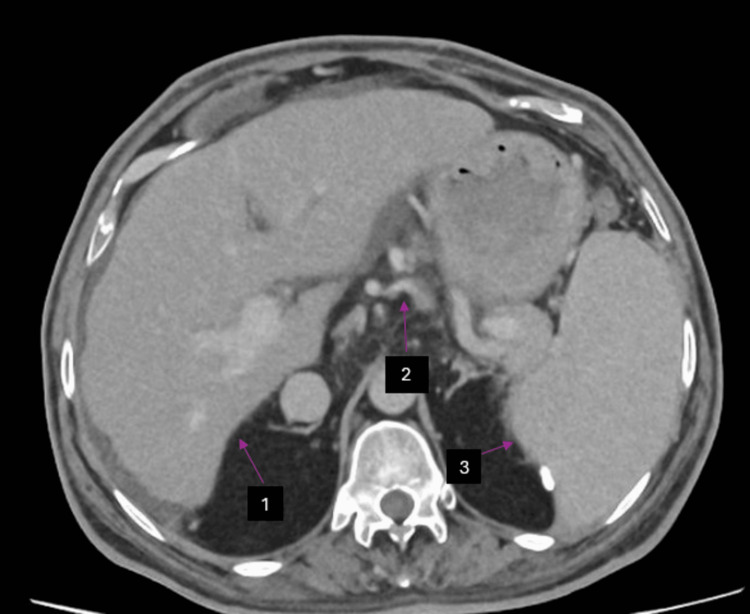
CT abdomen and pelvis Arrows indicating (1) cirrhotic liver, (2) collaterals indicating portal hypertension, and (3) splenomegaly

A contrast-enhanced computed tomography (CECT) of the chest and abdomen revealed an enlarged liver with an irregular outline and intra-abdominal collaterals, further confirming portal hypertension (Figure 4). 

This case was then discussed in the Upper Gastrointestinal multidisciplinary team where treatment options were explored. Given the presence of portal hypertension and oesophageal varices, post-operative mortality risk was high [1-4], and the patient was deemed unfit for surgical intervention. A decision to treat his achalasia with BTI into the LOS was made [5]. This was done under the EUS guidance to avoid injecting the botulinum toxin into any intervening vessels or varices [6,7]. The patient's symptoms improved dramatically after this treatment, and he was referred for liver transplant workup. 

## Discussion

Achalasia is a relatively rare oesophageal motility disorder which manifests with dysphagia, regurgitation, chest discomfort, and weight loss. The coexistence of achalasia and oesophageal varices is extremely rare, with an international study quoting around 12 reported cases in the literature, with only eight receiving treatment [1]. Although the exact pathophysiology remains unclear, the aetiology of achalasia is multifactorial, involving immune-mediated inflammation of ganglion cells within the oesophagus. This leads to loss of inhibitory neurons in the myenteric plexus of the LOS and oesophageal wall, which leads to decreased relaxation of the LOS and aperistalsis. Standard treatments for achalasia, such as POEM or pneumatic dilation, carry risks of perforation and bleeding. In patients with varices, these risks are amplified due to the potential for variceal bleeding [2,7]. 

The diagnostic workup consists of a barium swallow (Figure 2), OGD, and high-resolution oesophageal manometry (Figures 1, 3). While the OGD and barium swallow study remain essential for ruling out anatomical abnormalities, they have limited sensitivity in diagnosing achalasia [10]. 

The treatment includes medical, endoscopic, and surgical approaches [1,6]. Medical therapy is typically considered only in specific circumstances. It is usually reserved for patients who are not suitable candidates for more definitive treatments like pneumatic dilation, POEM, or surgical myotomy due to advanced age, significant comorbidities, or other factors that increase surgical risk [11]. Additionally, it might be used as a temporary measure to alleviate symptoms while awaiting more invasive procedures or if other treatments like Botox injections have not been effective. Hence, medical management is a less invasive and less effective alternative for a select group of patients, rather than a primary long-term solution [8,10].

In the presence of oesophageal varices, the treatment of achalasia cardia could lead to complications such as bleeding of the oesophageal varices [2,7]. On the other hand, treating oesophageal varices in the presence of achalasia cardia with banding could lead to worsening oesophageal motility and hence worsening of symptoms related to achalasia [11]. In these cases, it is important to consider a multidisciplinary team approach regarding the management of achalasia. Usually, a safer approach is to inject botulinum toxin into the LOS under EUS guidance to avoid injecting it into the intervening varices [6,7,10].

## Conclusions

The coexistence of achalasia and oesophageal varices is a rare clinical scenario, presenting significant management challenges. Myotomy is often precluded in patients who are poor surgical candidates due to underlying cirrhosis, while pneumatic dilation carries a high risk of precipitating significant bleeding from fragile oesophageal varices.

In light of these complexities, BTI into the LOS under EUS guidance emerges as an attractive and safer alternative for these high-risk patients. While the effects of botulinum toxin are temporary, typically lasting between six months and two years, its lower invasiveness makes it a valuable option for symptom relief, without exacerbating the risk of variceal bleeding. Given the rarity and inherent challenges of this condition, a multidisciplinary team approach is crucial. Such an approach carefully weighs the risks and benefits of treatment options, such as EUS-guided Botox injections, cautious pneumatic balloon dilation, or highly individualized surgical considerations, to ensure the best possible patient outcomes. Given the temporary effects of botulinum toxin therapy, continuous follow-up and symptom monitoring are crucial to adjust treatment strategies as needed to manage long-term outcomes effectively.

## References

[1] Pesce M, Magee C, Holloway RH (2019). The treatment of achalasia patients with esophageal varices: an international study. United Eur Gastroenterol J.

[2] Lozano-Lanagrán M, Lavín-Castejón I, Alcaín-Martínez G (2011). Treatment of achalasia with botulinum toxin injection guided by endoscopic ultrasonography in a patient with esophageal varices. Rev Esp Enferm Dig.

[3] Rana SS, Bhasin DK, Rao C, Sarwal R, Singh K (2013). Achalasia cardia associated with esophageal varices: a therapeutic dilemma. Ann Gastroenterol.

[4] Pinillos H, Legnani P, Schiano T (2006). Achalasia in a patient with gastroesophageal varices: problematic treatment decisions. Dig Dis Sci.

[5] Lakhtakia S, Monga A, Gupta R (2011). Achalasia cardia with esophageal varix managed with endoscopic ultrasound-guided botulinum toxin injection. Indian J Gastroenterol.

[6] Désilets E, Belle A, Boustière C, Laquière A (2016). Pneumatic dilation for achalasia in a patient with esophageal varices. Endosc Int Open.

[7] Momodu II, Wallen JM (2023). Achalasia. StatPearls [Internet].

[8] Mittal R (2011). Motor function of pharynx, esophagus, and its sphincters. Integrated Systems Physiology: from Molecule to Function to Disease.

[9] Jabbari B (2024). Botulinum Toxin Treatment: What Everyone Should Know. Botulinum toxin treatment of achalasia.

[10] Sobotka LA, Ramsey ML, Wellner M, Kelly SG (2019). Rare cause of dysphagia after esophageal variceal banding: a case report. World J Gastrointest Endosc.

[11] Vaezi MF, Pandolfino JE, Yadlapati RH, Greer KB, Kavitt RT (2020). ACG clinical guidelines: diagnosis and management of achalasia. Am J Gastroenterol.

[12] Yadlapati R, Kahrilas PJ, Fox MR (2021). Esophageal motility disorders on high-resolution manometry: Chicago classification version 4.0(©). Neurogastroenterol Motil.

